# Exciting new advances in oral cancer diagnosis: avenues to early detection

**DOI:** 10.1186/1758-3284-3-33

**Published:** 2011-07-28

**Authors:** Ravi Mehrotra, Dwijendra K Gupta

**Affiliations:** 1 Department of Pathology, Moti Lal Nehru Medical College, Lowther Road Allahabad, 211001 India; 2 Department of Biochemistry and Coordinator-Chair, Center of Bioinformatics, University of Allahabad, Allahabad, 211001 India; 3 Present Address: Department of Biochemistry, University of Bologna, Italy

**Keywords:** Oral Cancer, Diagnosis, Brush Biopsy, DNA, Saliva, Biomarkers, Spectroscopy

## Abstract

The prognosis for patients with oral squamous cell carcinoma remains poor in spite of advances in therapy of many other malignancies. Early diagnosis and treatment remains the key to improved patient survival. Because the scalpel biopsy for diagnosis is invasive and has potential morbidity, it is reserved for evaluating highly suspicious lesions and not for the majority of oral lesions which are clinically not suspicious. Furthermore, scalpel biopsy has significant interobserver and intraobserver variability in the histologic diagnosis of dysplasia. There is an urgent need to devise critical diagnostic tools for early detection of oral dysplasia and malignancy that are practical, noninvasive and can be easily performed in an out-patient set-up. Diagnostic tests for early detection include brush biopsy, toluidine blue staining, autofluorescence, salivary proteomics, DNA analysis, biomarkers and spectroscopy. This state of the art review critically examines these tests and assesses their value in identifying oral squamous cell carcinoma and its precursor lesions.

## Introduction

In a recent report from the American Cancer Society, it was estimated that 36,540 new cases of oral cavity and pharyngeal malignancies are likely to be diagnosed in the United States during 2010, and 7,880 patients will die of the disease. In developed countries like the United States, the five year survival was 63% between 1999-2005 - an increase from 53% during the time period from 1975-77; this difference was found to be statistically significant[1]. The improved survival rates may be partially explained by the increasing use of newer diagnostic modalities that detect the disease in its precursor stage and/or use of newer chemotherapeutic options.

### Approaches to Early Detection of Dysplasia and Oral Cancer

There are two approaches in the early detection of oral dysplasia and cancer: 1) oral cancer screening programs that identify asymptomatic patients with suspicious lesions and 2) employing specific diagnostic tools to identify dysplasia and early oral cancers in asymptomatic patients with an oral abnormality. The benefits and limitations of these approaches will be addressed in this review.

### Oral Cancer Screening

Screening for oral cancer implies searching for oral precancerous and cancerous lesions, typically before symptoms occur. A number of established cancer screening programs for a variety of malignancies have been shown to significantly reduce patient morbidity and mortality - including the Pap test for cervical cancer and mammography for breast cancer. However, several publications have demonstrated that oral cancer screening has limited value as a method for detecting precancerous or early cancerous lesions. In the only randomized controlled oral cancer screening trial conducted in India and involving over 130,000 individuals, the authors concluded that visual examination was useful as a method of screening for oral cancer only in high risk cases like chronic smokers or alcoholics [2].

Oral cancer screening is fraught with problems including the fact that approximately 5-15% of the general population may have an oral mucosal lesion. While the majority of these lesions are benign, clinical inspection alone cannot differentiate which lesions are potentially precancerous and cancerous and which ones are benign. The classic clinical presentation of a premalignant lesion or malignancy includes a red spot, white spot or persistent ulcer. However, only a small percentage of these types of lesions are cancerous and an oral examination unfortunately cannot discriminate between lesions that are potentially dangerous from lesions that are benign. A Cochrane review on this subject failed to find any evidence to confirm or refute the usefulness of screening for oral malignancies [3].

### Early Diagnosis

Early detection of oral cancer is one of the most efficient ways to reduce the high mortality from this disease. Early detection can minimize the morbidity of the disease and its treatment, which is associated with a severe loss of function, disfigurement, depression and poor quality of life.

However, based upon the National Cancer Institute's SEER program, which collects data on oral cancer, there has been little or no change in the past twenty years in the detection of oral cancers at early stages [4]. Unfortunately, most patients are diagnosed with advanced stage disease.

Early detection therefore necessitates raising awareness in the general public and improving access to oral health services for all segments of the population.

Oral squamous cell carcinoma (OSCC) is almost always preceded by a visible precancerous lesion-dysplasia. As highlighted by the American Dental Association, "Identifying white and red the spots that show dysplasia and removing them before they become cancer has proved to be one of the most effective methods for reducing the incidence and mortality of cancer" [5]. Malignant transformation of dysplasia, which is quite unpredictable, occurs over years - during which time the lesion can be treated, potentially preventing oral cancer from developing. Oral precancerous lesions may also occasionally regress if the healthcare professional motivates the patient to reduce the risk factors including elimination of carcinogens including tobacco and alcohol.

### Light-based oral cancer screening aids

A number of light-based oral cancer screening aids have been developed and aimed at assisting in the identification of precancerous and cancerous lesions at their earliest stage. (Table 1) Specifically, these aids are intended to be used as adjuncts to the conventional oral cavity examination to help visualize lesions. Vizilite Plus with TBlue system (Zila Pharmaceuticals, Phoenix, Arizona, U.S.), VELscope (LED Dental, White Rock, British Columbia, Canada) Microlux/DL (AdDent Inc, Danbury, Connecticut) and Orascoptic DK (Orascoptic, Middleton, WI) are commercially available light-based systems that are based upon the assumption that abnormal metabolic or structural changes have different absorbance and reflectance properties.

**Table 1:** The various diagnostic modalities for oral cancer detection.

Visual examination
Excision biopsy and Histopathology
Oral brush biopsy (OralCDx)
Toluidine blue
Light-based detection systems
Chemiluminescence (ViziLite Plus; Microlux/DL, Orascoptic-DK)
Tissue fluorescence imaging (VELscope)
Tissue fluorescence spectroscopy
Biomarkers
DNA-analysis
Laser capture microdissection

**Figure 1:**
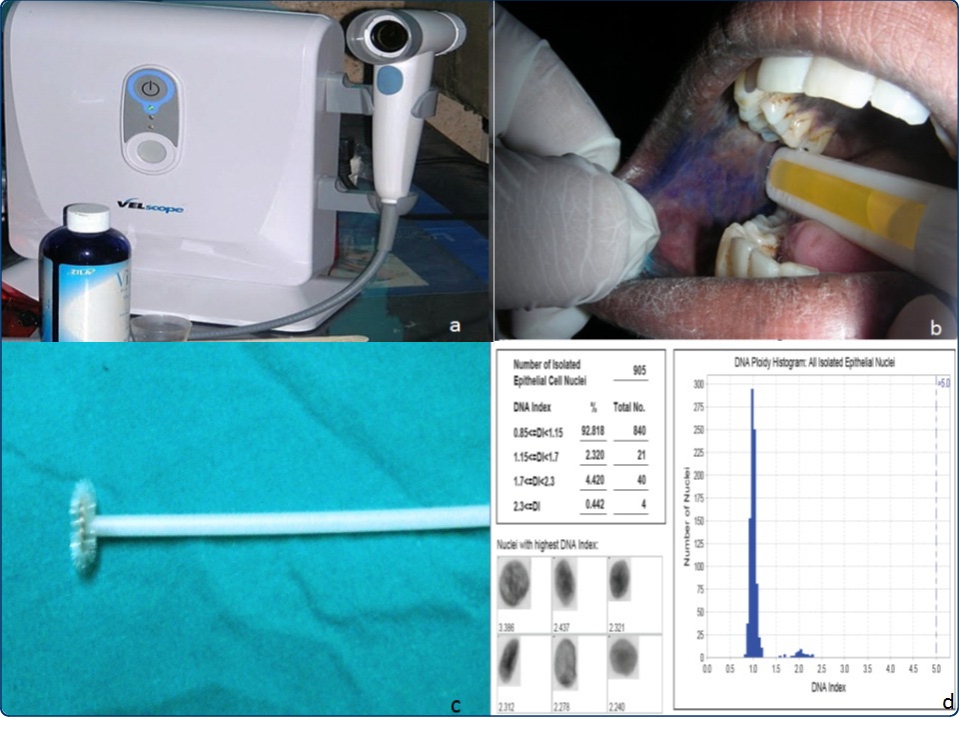
**a) Velscope, b) Vizylite with toluidine blue staining, c) Patented brush used for oral brush biopsy test, d) Automated DNA ploidy**.

VELscope is a handheld device that uses visible light in the 430 nm wavelength in order to cause fluorescent excitation of certain compounds in the tissues. (Figure 1a) With Vizilite, patients' first rinse with acetic acid and then the oral cavity is examined with an illuminated chemiluminescent light stick. (Figure 1b) Microlux is similar to Vizilite and requires the patient first rinse with acetic acid; the oral cavity is then examined with a battery-powered fiberoptic visible light source instead of a chemiluminescent visible light source. Orascoptic DK also requires an acetic acid rinse and uses a three-in-one device that employs a battery-powered handheld light source. Since none of these medical devices is a diagnostic test, the manufacturer of these screening aids do not make any claim that the device is either sensitive or specific to the identification of any type of abnormal oral lesion. Furthermore, as we reported in a prospective study examining the potential benefits of several of these lights, the sensitivity of Vizilite was 0% and the sensitivity of VELscope was also poor-50%. We concluded that the use of ViziLite or VELscope along with a conventional screening examination was not beneficial in identifying dysplasia or cancer and clinicians/and patients could have a false sense of security after obtaining a negative ViziLite or VELscope examination result because potentially large numbers of precancerous and cancerous lesions would be missed by both. Until additional studies are performed, these screening lights should only be used to help identify lesions that may have been overlooked with a conventional oral examination and not for determining whether a lesion is precancerous or cancerous. Only a definitive test examining cells or tissue can determine the biologic behavior of a lesion [6].

## Diagnostic Tests

### Cytological Techniques

During the last few decades, oral cytology has resurfaced as the focus of scientific research. However, in contrast to the sampling of cells of the uterine cervix, analysis of surface epithelial cells of the oral cavity and oropharynx by standard exfoliative cytology has proven unreliable so far. The shape of the oral cavity makes it impossible to examine the complete mucosal surface. Without loss of minimal invasiveness, it was not possible to access the deeper cell layers of the oral cavity with conventional exfoliative cytology[7].

### Brush Biopsy

During the 1980s, a brush was introduced for cervical smears in gynecological lesions and was later modified for oral smears too. (Figure 1c) This technique demonstrated better cell spreading on objective slides compared with smears obtained by using the conventional wooden spatula as well as an improvement in the cellular adequacy of the smears. The importance of oral brush biopsy was emphasized in a multicenter study where nearly 5% of clinically benign-appearing oral mucosal lesions were sampled by using this technique and later confirmed by using scalpel biopsy to represent dysplastic epithelial changes or invasive cancer [8].

OralCDx^® ^(OraCDx Laboratories, Inc. Suffern, NY), the oral brush biopsy with computer assisted analysis, is a diagnostic test that identifies dysplasia in common spots that often have no suspicious clinical features. Unlike exfoliative cytology, the brush biopsy collects cells from the full thickness of the oral epithelium. The brush biopsy is a chair-side, easy to perform, painless test that can be used to evaluate any suspicious lesion including common small white and red oral lesions to rule out dysplasia. Since most oral lesions are benign, most test results are likely to be benign. Approximately 10% of all cases usually turn out to be abnormal. Based upon the findings, the laboratory provides specific guidance on these abnormal cases sometimes recommending scalpel biopsy, retesting or observation.

The accuracy of the brush test has been the subject of many published studies. In every study in which an oral lesion was simultaneously tested with both a brush biopsy and scalpel biopsy, this test has been shown to have a sensitivity and specificity well over 90% [8,9]. These studies demonstrate that the brush biopsy has a high sensitivity in ruling out the presence of dysplasia and cancer making it a practical way to evaluate lesions without an obvious etiology such as infection of trauma.

Discrepancies of brush test and scalpel biopsy results have been reported anecdotally and have incorrectly been labeled brush "false negatives." Unfortunately these anecdotes been quoted repeatedly in the literature despite the fact that they have no validity at all. These discrepant results were all from cases where the scalpel biopsy was performed months after from the brush biopsy. Within a given oral lesion, dysplasia is multicentric and unless the 2 biopsy samples happen to sample the same part of the dysplastic lesion, the results will be discrepant. Furthermore, the biologic nature of a lesion may change over time as benign lesions may become dysplastic and dysplasia may also regress. Most importantly, the histologic diagnosis of dysplasia is not easily reproduced amongst oral pathologists and therefore a discrepant result between brush biopsy and scalpel biopsy may in fact represent a false negative or false positive scalpel biopsy result. Therefore, when comparisons are made between any 2 biopsy techniques (i.e. brush biopsy vs. scalpel biopsy or scalpel biopsy vs. scalpel biopsy) the only valid studies are those which compare the results of both biopsies performed at the same time and from the same portion of the suspicious lesion.

To help localize the optimal site for brushing an abnormality, Gupta et al combined conventional oral brush biopsy with the application of toluidine blue to localize suspect mucosal areas [10].

### Scalpel biopsy

Until now, tissue sampling by scalpel biopsy and subsequent histological examination have been the cornerstone for diagnosing premalignant and malignant oral diseases. Unfortunately, scalpel biopsy has many inherent limitations which health care practitioners need to be aware of. For example, vague histopathological definitions as well as histological misinterpretation resulting in false negatives and false positives should be kept in mind while interpreting the results of scalpel biopsies. An oral biopsy is invasiveand involves both psychological implications for the patient as well as possible often technical difficulties for the health practitioner. When lesions are extensive, the most representative areas must be selected to avoid diagnostic errors[11].

Oral biopsy specimens can be affected by a number of artefacts resulting from crushing, fulguration or incorrect fixation and freezing[12]. There is a long standing controversy regarding selection both of the technique (incisional versus excisional) and of the surgical instruments used to avoid artefacts; punch biopsy may have some benefits [13].

Experimental studies have detected an increased frequency of neck metastasis from stage I and stage II OSCC after incisional biopsy and the presence of tumour cells have been noticed in the peripheral blood 15 min after incisional biopsies using a conventional scalpel[14,15]. Consequently, it has been hypothesized that the use of a laser beam to obtain biopsy material could minimize seeding of cells but studies are needed to support this theory[16].

The limitations of comparing 2 biopsy results performed at different time has been highlighted in a study of 200 patients with leukoplakia, who underwent scalpel biopsies at different times with an agreement rate between two scalpel biopsies of only 56%. In addition, clinicians should be aware of possible under diagnosis after incisional biopsy-particularly in cases of "hybrid" forms of OSCC and non-homogeneous lesions, depending on the site where the biopsy is performed[17,18].

A number of histological characteristics of the primary tumour, such as the grade of malignancy and depth of invasion, have been shown to have prognostic value in terms of tumour recurrence, lymph node involvement, and cause-specific survival hence, it is important for an incisional biopsy to be of sufficient size and depth to include part of the advancing margin of tumour[19]. Ideally, the deep margin should be included, but if this is not possible (e.g., in large tumours), the peripheral margin is often sufficiently representative to allow provisional assessment.

Finally, a high inter- and intraobserver variability in histologically diagnosing dysplasia has been described by many authors. The difficulty of accurately diagnosing dysplasia and reproducing those results can have significant implications for patients who are provided with false positive and false negative scalpel biopsy results. As already highlighted in this review, when two scalpel biopsies are performed at different times by different examiners, the agreement rate between them was only 56%[17]. It is evident that even incisional biopsies of suspicious lesions which have a limited reproducibility may, at times, result in a more or less aggressive surgical and/or radio-chemotherapeutic approach[11].

## Experimental Screening AIDS and Diagnostic Tests in Development

### Vital staining

Toluidine blue (TB) staining is claimed to be a simple, inexpensive and sensitive adjunct tool for identifying early OSCC and high-grade dysplasias [20]. When a 1% aqueous TB solution is applied to a suspicious lesion for 30 seconds, this acidophilic metachromatic nuclear stain helps to differentiate areas of carcinoma in situ or invasive carcinoma from normal tissue. Although TB has been found to be highly sensitive and moderately specific for malignant lesions, it is far less sensitive for premalignant lesions with false negative rates of up to 58% reported for identifying mild-to-moderate dysplasia [10,21].

Toluidine blue has also been demonstrated to help assess the status of margins around oral cancer at the time of resection [22]. Although toluidine blue test is helpful in identifying oral cancers, it should not be viewed as a substitute for biopsy, and a negative test does not preclude the presence of dysplasia or even oral cancer.

## Laser Capture Microdissection

Laser capture microdissection (LCM) has made the study of cancer biology more precise and has greatly boosted the efforts in defining the molecular basis of malignancy [23]. LCM provides an ideal method for the extraction of cells from specimens in which the exact morphology of both the captured cells and the surrounding tissue are preserved. When rapid immunohistochemical staining techniques are combined with LCM, more accurate microdissection of cellular subsets can be obtained [24]. LCM may be also used to detect the biomarkers and establish protein fingerprint models for early detection of OSCC. LCM combined with SELDI-TOF-MS technology and bioinformatics approaches may not only facilitate the discovery of better biomarkers but also provide a useful tool for molecular diagnosis [25,26].

## Dna-Analysis

DNA image cytometry measures ploidy status to determine the malignant potential of cells. After staining with Feulgen dye, the cytological samples are compared with a reference group of cells. A computer-assisted analysis has been recently designed to identify deviations of cellular DNA content. Genomic instability contributes towards cancer development, and abnormal DNA content may distinguish the dysplastic lesions that might progress to cancer (Figure 1d) [27]. Several studies confirm the usefulness of DNA ploidy analysis as an adjunct to conventional cytology assessment of cytobrush samples for detection of oral cancer [27-30]. An increase in sensitivity and specificity of oral brush biopsy to 100% has been reported [29,31]. Multimodal cell analysis (MMCA) and mechanical phenotyping have been used for early detection of oral malignancies [32,33].

Oral brush samples were examined to detect non-diploid cells (NDC) combined with morphological and cytogenetic analysis. It has been suggested that the combined morphological and cytogenetic analysis of cells collected by a non-invasive brush may enable early detection of potentially malignant cells [34]. In a study, Bremmer et al have investigated allelic imbalance in brushes from 25 patients with leukoplakia and reported genetic changes in 40% of these patients as compared to none in controls, yielding a sensitivity of 78% and positive predictive value of 100% [35].

## Saliva-Based Oral Cancer Diagnosis

Saliva testing, a non-invasive alternative to serum testing, may be an effective modality for diagnosis, determining prognosis of oral cancer and for monitoring post-therapy status. Saliva, as a diagnostic tool, has many advantages over serum, aside from the ability of being collected non-invasively. Saliva may provide a cost-effective and practical approach for the screening of large populations. It may be used to measure specific salivary macromolecules as well as examining proteomic or genomic targets such as enzymes, cytokines, growth factors, metalloproteinases, endothelin, telomerase, cytokeratins, mRNAs and DNA transcripts [36-38].

The six most studied epithelial serum circulatory tumor markers in the saliva of carcinoma patients are Cyfra 21-1, TPS, carcinoembryonic antigen (CEA), SCC, CA125, and CA19-9. Significant increase (of 400%) in salivary concentrations of Cyfra 21-1, TPS and CA125 were shown with sensitivity, specificity, and negative and positive predictive values of 71%, 75%, 71%, and 75%, respectively. On the other hand CEA, SCC and CA19-9, did not reach statistical significance[30-41]. CD44, a multi-structural and multifunctional cell surface transmembrane glycoprotein molecule has also been detected in saliva[42].

## Lab-on-a-Chip

Broadly, microfluidics technology -also referred to as lab-on-a-chip or micro-total-analysis systems (TAS)-is the adaptation, miniaturization, integration, and automation of analytical laboratory procedures into a single device or "chip." Microfluidics is often regarded as the chemistry or biotechnology equivalent of the silicon integrated circuit chip that has revolutionized electronics, computers, and communications. The detection of oral dysplastic and cancer cells within the chip utilizes membrane-associated cell proteins that are singularly expressed on the cell membranes of dysplastic and cancer cells as well as their unique gene transcription profiles [43].

## Microscopy

Spectral cytopathology (SCP) is a recently developed technique for diagnostic differentiation of disease in individual exfoliated cells. Papamarkakis et al carried out SCP by collecting information on each cell's biochemical composition through infrared micro-spectral measurement, followed by multivariate data analysis. Deviations from a cell's natural composition produced specific spectral patterns that were exclusive to the cause of the deviation or disease. These unique spectral patterns were reproducible and analyzed through multivariate statistical methods to detect cells in dysplasia, neoplasia, or viral infection at the molecular level [44].

Multispectral digital microscope (MDM) has also been utilized as a tool to improve detection of oral neoplasia. MDM acquires *in-vivo *images in different modes i.e. fluorescence, narrow-band (NB) reflectance, and orthogonal polarized reflectance (OPR) to enable evaluation of lesions [45].

Poh *et al *studied 122 oral mucosal biopsies, 20 surgical specimens and assessed the fluorescence visualization (FV) status, histology and loss of heterozygosity. They concluded that direct visualization of subclinical field changes around oral cancers, documenting alterations in fluorescence and direct FV can identify subclinical high-risk fields with cancerous and precancerous changes in the operating room setting[46].

## Spectroscopy

Autofluorescence and chemiluminescencehave been studied as non-invasive in-vivo tools for the detection of (pre-)malignant tissue alterations. Autofluorescence of tissues under excitation with light is produced by several endogenous fluorophores. It was reported that autofluorescence spectroscopy may provide valuable information for diagnosis and monitoring the therapeutic response in oral submucous fibrosisand should be validated with more studies involving large samples and longer follow-up[47,48].

The wavelength of absorption is affected by changes in blood content and oxygenation, which are the signs of disease due to altered tissue metabolism or neovascularization. Diffuse reflectance spectroscopy (DRS) has been studied as a non-invasive in-vivo tool for the detection of (pre)malignant tissue alterations[49].

In another study, the DRS ratio of oxygenated hemoglobin bands at 545 and 575 nm was used for grading of malignancy. A sensitivity of 100% and specificity of 86% for differentiating dysplasia from hyperplasia, and a sensitivity of 97% and specificity of 86% for discriminating hyperplasia from normal was obtained [50]. Pavlovaet al reported that examining oral lesions with optical tools may result in a loss of fluorescence intensity and may fail to distinguish benign from precancerous lesions[51].

## Tomography

Optical coherence tomography (OCT)is a non-invasive tomographic imaging modality to detect areas of inflammation, dysplasia and cancer. OCT records subsurface reflections to build a cross-sectional architectural image of tissue. Contrast in these images may be enhanced by utilizing surface plasmon resonant gold nanoparticles. It was concluded that this multimodal delivery of antibody-conjugated Polyethylene glycol linked gold nanoparticles enhances the contrast in *in-vivo *OCT images of oral dysplasia in a hamster model [52]. A recent pilot study in 27 patients confirmed the feasibility of using OCT to identify architectural changes in malignant cells but unfortunately, it could not provide a diagnosis or differentiate between lesions [53].

## Conclusions

Early diagnosis of oral cancer is a priority health objective, in which oral health professionals may play a pivotal role. Detection should lead to less damage from cancer therapy and to a better prognosis. There are also a number of novel techniques that may variously help in the diagnosis of oral malignancy. Lately, light-based detection systems have been claimed to improve sensitivity and specificity, but so far, controlled studies have failed to justify their application. Brush biopsy and scalpel biopsy are effective diagnostic tests for evaluating suspicious oral lesions which may be precancerous or cancerous. Light based screening aids should only be employed as an adjunct to the clinical examination for identifying oral lesions that may have been overlooked with a conventional oral examination and not for determining the biologic nature of a lesion. However, controlled trials in both high and low risk populations with histologic outcomes and critical appraisal from the medical community are required before they can be integrated into practice.

## Competing interests

The authors declare that they have no competing interests.

## Authors' contributions

RM conceived and researched the manuscript; DKG reviewed and finalized the manuscript.

## Authors' Information

1. Ravi Mehrotra MD,Ph.D,MIAC, Professor of Pathology, Division of Cytopathology, Moti Lal Nehru Medical College.

2. Dwijendra K. Gupta,Ph.D.   Professor of Biochemistry and   Coordinator-Chair, Center of Bioinformatics, Institute of Interdisciplinary Studies, University of Allahabad, Allahabad India - Presently at University of Bologna, Italy

## References

[B1] Jemal A Siegel R Xu J Ward E Cancer Statistics 2010 CA Cancer J Clin 2010 60 277 300 10.3322/caac.20073 20610543

[B2] Sankaranarayanan R Ramadas K Thomas G Muwonge R Thara S Mathew B Rajan B Trivandrum Oral Cancer Screening Study Group. Effect of screening on oral cancer mortality in Kerala, India: a cluster-randomised controlled trial Lancet 2005 365 9475 1927 33 10.1016/S0140-6736(05)66658-5 15936419

[B3] Kujan O Glenny AM Oliver RJ Thakker N Sloan P Screening programmes for the early detection and prevention of oral cancer Cochrane Database Syst Rev 2006 3 CD004150 16856035 10.1002/14651858.CD004150.pub2

[B4] SEER Cancer Statistics Review, 1975-2005, National Cancer Institute 2008 http://seer.cancer.gov/csr/1975_2005/based on November 2007 SEER data submission, posted to the SEER web site

[B5] http://www.ada.org/2607.aspx

[B6] Mehrotra R Hullmann M Smeets R Reichert TE Driemel O Oral cytology revisited J Oral Pathol Med 2009 38 161 6 19213102 10.1111/j.1600-0714.2008.00709.x

[B7] Mehrotra R Singh M Thomas S Nair P Pandya S Nigam NS Shukla P Cross-sectional study evaluating chemiluminescence and autofluorescence in the detection of clinically innocuous precancerous and cancerous oral lesions J Am Dent Assoc 2010 141 151 6 20123872 10.14219/jada.archive.2010.0132

[B8] Sciubba JJ Improving detection of precancerous and cancerous oral lesions. Computer-assisted analysis of the oral brush biopsy J Am Dent Assoc 1999 130 1445 57 10570588 10.14219/jada.archive.1999.0055

[B9] Scheifele C Schmidt-Westhausen AM Dietrich T Reichart A The sensitivity and specificity of the oral CDx technique: evaluation of 103 cases Oral Oncol 2004 40 824 8 10.1016/j.oraloncology.2004.02.004 15288838

[B10] Gupta A Singh M Ibrahim R Mehrotra R Utility of toluidine blue and oral brush biopsy in oral precancerous lesions and squamous cell carcinoma Acta Cytol 2007 51 788 94 10.1159/000325843 17910350

[B11] Holmstrup P Vedtofte P Reibel J Stoltze K Oral premalignant lesions: is biopsy reliable? J Oral Path Med 2007 36 262 6 10.1111/j.1600-0714.2007.00513.x 17448135

[B12] Seoane J Varela-Centelles P Ramirez JR Romero MA De La Cruz A Artefacts produced by suture traction during incisional biopsy of oral lesions Clin Otolaryngol 2002 27 549 53 10.1046/j.1365-2273.2002.00619.x 12472529

[B13] Moule L Parsons PA Irvine GH Avoiding artefacts in oral biopsies: the punch biopsy versus the incisional biopsy Br J Maxillofac Surg 1995 33 244 7 10.1016/0266-4356(95)90010-1 8736752

[B14] Kusukawa J Suefuji Y Ryu F Noguchi R Iwamoto O Kameyama T Dissemination of cancer cells into circulation occurs by incisional biopsy of oral squamous cell carcinoma J Oral Pathol Med 2000 29 303 7 10.1034/j.1600-0714.2000.290703.x 10947245

[B15] Dyavanagoudar S Kale A Bhat K Hallikerimath S Reverse transcriptase polymerase chain reaction study to evaluate dissemination of cancer cells into circulation after incision biopsy in oral squamous cell carcinoma Indian J Dent Res 2008 19 315 9 10.4103/0970-9290.44534 19075434

[B16] Klein DR The use of the carbon dioxide laser in plastic surgery South Med J 1977 70 429 31 10.1097/00007611-197704000-00018 322307

[B17] Lee JJ Hung HC Cheng SJ Chiang CP Liu BY Yu CH Jeng JH Chang HH Kok SH Factors associated with underdiagnosis from incisional biopsy of oral leukoplakic lesions Oral Surg Oral Med Oral Pathol Oral Radiol Endod 2007 104 2 217 25 10.1016/j.tripleo.2007.02.012 17560138

[B18] Woolgar JA Triantafyllou A Pitfalls and procedures in the histopathological diagnosis of oral and oropharyngeal squamous cell carcinoma and a review of the role of pathology in prognosis Oral Oncol 2009 45 361 85 10.1016/j.oraloncology.2008.07.016 18849188

[B19] Bryne M Koppang H Lilleng R Kjaerhheim A Malignancy trading of the deep invasive margins of oral squamous cell carcinomas has high prognostic value J Pathol 1992 166 375 81 10.1002/path.1711660409 1517891

[B20] Mashberg A Toluidine blue J Can Dent Assoc 1995 61 11 922 944 8521318

[B21] Martin IC Kerawala CJ Reed M The application of toluidine blue as a diagnostic adjunct in the detection of epithelial dysplasia Oral Surg Oral Med Oral Pathol Oral Radiol Endod 1998 85 4 444 6 10.1016/S1079-2104(98)90071-3 9574954

[B22] Epstein JB Güneri P The adjunctive role of toluidine blue in detection of oral premalignant and malignant lesions Curr Opin Otolaryngol Head Neck Surg 2009 17 2 79 87 10.1097/MOO.0b013e32832771da 19374030

[B23] Mehrotra R Gupta A Singh M Ibrahim R Application of cytology and molecular biology in diagnosing premalignant or malignant oral lesions Mol Cancer 2006 5 1 11 16556320 10.1186/1476-4598-5-11 PMC1448188

[B24] Fend F Emmert-Buck MR Chuaqui R Cole K Lee J Liotta LA Raffeld M Immuno-LCM: Laser capture micro dissection of immunostained frozen sections for mRNA analysis Am J Pathol 1999 154 61 6 10.1016/S0002-9440(10)65251-0 9916919 PMC1853427

[B25] He H Sun G Ping F Laser-capture microdissection and protein extraction for protein fingerprint of OSCC and OLK Artif Cells Blood Substit Immobil Biotechnol 2009 37 5 208 13 10.1080/10731190903199028 19735007

[B26] Mehrotra R Vasstrand EN Ibrahim SO Recent advances in understanding carcinogenicity of oral squamous cell carcinoma: from basic molecular biology to latest genomic and proteomic findings Cancer Genomics Proteomics 2004 1 283 94 31394607

[B27] Bradley G Odell EW Raphael S Ho J Le LW Benchimol S Kamel-Reid S Abnormal DNA content in oral epithelial dysplasia is associated with increased risk of progression to carcinoma Br J Cancer 2010 103 9 1432 42 10.1038/sj.bjc.6605905 20859287 PMC2990600

[B28] Maraki D Becker J Boecking A Cytologic and DNA cytometric very early diagnosis of oral cancer J Oral Pathol Med 2004 33 398 404 10.1111/j.1600-0714.2004.0235.x 15250831

[B29] Maraki D Yalcinkaya S Pomjanski N Megahed M Boecking A Becker J Cytologic and DNA-cytometric examination of oral lesions in lichen planus J Oral Pathol Med 2006 35 4 227 32 10.1111/j.1600-0714.2006.00401.x 16519770

[B30] Handschel J Oz D Pomjanski N Depprich R Ommerborn MA Braunstein S Kübler NR Meyer U Böcking A Additional use of DNA-image cytometry improves the assessment of resection margins J Oral Pathol Med 2007 36 8 472 5 10.1111/j.1600-0714.2007.00564.x 17686005

[B31] Remmerbach TW Mathes SN Weidenbach H Hemprich A Böcking A Noninvasive brush biopsy as an innovative tool for early detection of oral carcinomas Mund Kiefer Gesichtschir 2004 8 4 229 36 15293118 10.1007/s10006-004-0542-z

[B32] Remmerbach TW Meyer-Ebrecht D Aach T Würflinger T Bell AA Schneider TE Nietzke N Frerich B Böcking A Toward a multimodal cell analysis of brush biopsies for the early detection of oral cancer Cancer Cytopathol 2009 117 3 228 35 10.1002/cncy.20028 19373897

[B33] Remmerbach TW Wottawah F Dietrich J Lincoln B Wittekind C Guck J Oral cancer diagnosis by mechanical phenotyping Cancer Res 2009 69 5 1728 32 10.1158/0008-5472.CAN-08-4073 19223529

[B34] Hirshberg A Yarom N Amariglio N Yahalom R Adam I Stanchescu R Ben-Dov I Taicher S Rechavi G Trakhtenbrot L Detection of non-diploid cells in premalignant and malignant oral lesions using combined morphological and FISH analysis - a new method for early detection of suspicious oral lesions Cancer Lett 2007 253 2 282 90 10.1016/j.canlet.2007.02.008 17386971

[B35] Bremmer JF Graveland AP Brink A Braakhuis BJ Kuik DJ Leemans CR Bloemena E van der Waal I Brakenhoff RH Screening for oral precancer with noninvasive genetic cytology Cancer Prev Res (Phila) 2009 2 2 128 33 10.1158/1940-6207.CAPR-08-0128 19174582

[B36] Nagler RM Saliva as a tool for oral cancer diagnosis and prognosis Oral Oncol 2009 45 1006 10 10.1016/j.oraloncology.2009.07.005 19828359

[B37] Bahar G Feinmesser R Popovtzer A Nagler RM Salivary analysis in oral cancer patients: DNA and protein oxidation, reactive nitrogen species, and antioxidant profile Cancer 2007 109 1 54 9 10.1002/cncr.22386 17099862

[B38] Li Y St John MA Zhou X Kim Y Sinha U Jordan RC Eisele D Abemayor E Elashoff D Park NH Wong DT Salivary transcriptome diagnostics for oral cancer detection Clin Cancer Res 2004 10 8442 50 10.1158/1078-0432.CCR-04-1167 15623624

[B39] Shpitzer T Bahar G Feinmesser R Nagler RM A comprehensive salivary analysis for oral cancer diagnosis J Cancer Res Clin Oncol 2007 133 9 613 7 10.1007/s00432-007-0207-z 17479291 PMC12160907

[B40] Zhong LP Zhang CP Zheng JW Li J Chen WT Zhang ZY Increased Cyfra 21-1 concentration in saliva from primary oral squamous cell carcinoma patients Arch Oral Biol 2007 52 11 1079 87 10.1016/j.archoralbio.2007.05.005 17612501

[B41] Zimmermann BG Wong DT Salivary mRNA targets for cancer diagnostics Oral Oncol 2008 44 5 425 9 10.1016/j.oraloncology.2007.09.009 18061522 PMC2408659

[B42] Screaton GR Bell MV Jackson DG Cornelis FB Gerth V Bell JI Genomic structure of DNA encoding the lymphocyte homing receptor CD44 reveals at least 12 alternatively spliced exons Proc Natl Acad Sci USA 1992 89 12160 4 10.1073/pnas.89.24.12160 1465456 PMC50718

[B43] Ziober BL Mauk MG Falls EM Chen Z Ziober AF Bau HH Lab-on-a-chip for oral cancer screening and diagnosis Head Neck 2008 30 1 111 21 10.1002/hed.20680 17902150

[B44] Papamarkakis K Bird B Bedrossian M Laver N Wein R Diem M Cytopathology by optical methods: spectral cytopathology of the oral mucosa Lab Invest 2010 90 4 589 98 10.1038/labinvest.2010.1 20142808 PMC2847622

[B45] Roblyer D Richards-Kortum R Sokolov K El-Naggar AK Williams MD Kurachi C Gillenwater AM Multispectral optical imaging device for in vivo detection of oral neoplasia J Biomed Opt 2008 13 2 024019 10.1117/1.2904658 18465982 PMC3970814

[B46] Poh CF Zhang L Anderson DW Durham JS Williams PM Priddy RW Berean KW Ng S Tseng OL MacAulay C Rosin MP Fluorescence visualization detection of field alterations in tumor margins of oral cancer patients Clin Cancer Res 2006 12 22 6716 22 10.1158/1078-0432.CCR-06-1317 17121891

[B47] Vedeswari CP Jayachandran S Ganesan S In vivo autofluorescence characteristics of pre- and post-treated oral submucous fibrosis: A pilot study Indian J Dent Res 2009 20 3 261 7 10.4103/0970-9290.57354 19884705

[B48] Kurachi C Fontana CR Rosa LE Bagnato VS Fluorescence spectroscopy for the detection of tongue carcinoma--validation in an animal model J Biomed Opt 2008 13 3 034018 10.1117/1.2937214 18601563

[B49] Majumder SK Majumder SK Ghosh N Kataria S Gupta PK Nonlinear pattern recognition for laser-induced fluorescence diagnosis of cancer Lasers Surg Med 2003 33 1 48 56 10.1002/lsm.10191 12866121

[B50] Mallia R Thomas SS Mathews A Kumar R Sebastian P Madhavan J Subhash N Oxygenated hemoglobin diffuse reflectance ratio for in vivo detection of oral pre-cancer J Biomed Opt 2008 13 4 041306 10.1117/1.2952007 19021314

[B51] Pavlova I Williams M El-Naggar A Richards-Kortum R Gillenwater A Understanding the biological basis of autofluorescence imaging for oral cancer detection: high-resolution fluorescence microscopy in viable tissue Clin Cancer Res 2008 14 8 2396 404 10.1158/1078-0432.CCR-07-1609 18413830 PMC2773159

[B52] Kim CS Wilder-Smith P Ahn YC Liaw LH Chen Z Kwon YJ Enhanced detection of early-stage oral cancer in vivo by optical coherence tomography using multimodal delivery of gold nanoparticles J Biomed Opt 2009 14 3 034008 10.1117/1.3130323 19566301 PMC2872553

[B53] Jerjes W Upile T Conn B Betz CS McKenzie G Radhi H Vourvachis M El Maaytah M Sandison A Jay A Hopper C In vitro examination of suspicious oral lesions using optical coherence tomography Br J Oral Maxillofac Surg 2010 48 18 25 10.1016/j.bjoms.2009.04.019 19726114

